# De La Lithontritie on Broiment De La Pierre Dans La Vessie

**Published:** 1829-02

**Authors:** 


### REVIEWS.

---

ART. III.—*De la Lithontritie ou Broiement de la pierre dans la Vessie; Par le* DOCTEUR CIVIALE. *Avec cinq plauches, pp.* 254, *A Paris* 1827.

*Lithontrity, or Breaking down the stone in the Bladder &c.*

Having been among the first in the United States, who were made acquainted with the ingenius inventions of Doctor Civiale, we feel a degree of self-reproach, at having so long deferred a notice of them. By the delay, however, we have come into possession of his book, from which and other documents—manuscript and printed—we hope to make some amends, both to him and our readers, for the procrastination. As far as we know, Dr. Samuel Brown, formerly professor of the Theory and Practice of Medicine in Transylvania University, was the first person who promulgated, in this country, a notice of Dr. C's practice. Being on a scientific visit to Paris, in the year 1824, on the 10th of August, he wrote to the Editor as follows:—

"I have had the uncommon good fortune to witness seven operations by Civiale, and have had every aid he could give me in order to make me master of it. It consists in reducing the stone in the bladder and taking it out without *shedding blood*. It is quite as painful to the *Surgeon* as to some of the *Patients*. They all laugh and talk during great part of the operation, and can all appear in the street the next day and often change their lodgings the same night. No one has died of it, or will probably die—I have seen it performed on a child nine years old, and on two men of seventy, who made very trifling complaint. Richerand attended with me yesterday morning and expressed the highest approbation; Percy and Chaussiere have done so in print, and Surgeons of great note from Russia, Poland, Vienna, Berlin &c. have assured me of their warmest approbation. Some of the great Surgeons, the High Priests and Scribes, object, and still offer victims to Moloch. I am satisfied, and can satisfy you. This is worth my voyage to Europe. Dumas had commenced dissolving the calculus by Galvanism, as I had proposed last year, and was succeeding; but he assured me that he thought Civiale had rendered all attempts of that kind unnecessary. This great improvement is in part attributable to Amusat's anatomical discovery, relative to the Urethra."

As Dr. Brown was reputed to be a sort of enthusiast, in regard to discoveries and improvements in the profession, his estimate of the Lithontrity of Dr. C. was received with some distrust, by many American Surgeons; and these doubts were not a little confirmed, by the trials which were soon after made by several surgeons in our great professional metropolis. On his return, he brought over drawings of the principal instruments used by Dr. C., copies of which were put into the hands of Mr. Lukens of that city. But they seem not to have been very accurate; and, in addition, that ingenious artist conceived, that certain variations might be made in their construction. This was accordingly done, and it was with these new tools, that American Surgery went to work. The results, we believe, were not encouraging; and although no people are more ready to adopt improvements—either real or pretended—than the American, Lithontrity has not as yet found any practical advocate among us. Meanwhile it has been practised, not only in Paris but other cities of France, with great success; and among other recent testimonies, in its favour we may refer again to that of Dr. Brown; who, on his late return from a second visit to Europe, assured us in conversation, that he had again witnessed several successful operations by Dr. C. and that the merits of Lithontrity were becoming generally acknowledged in France. The opinions and observations of our friend Dr. La Roche, one of the Editors, and at present a correspondent, of the highly respectable North American Medical and Surgical Journal, are to the same point. On the 11th of September, 1828, he writes, from Paris to his coadjutors, in the following language:—

"Having myself seen M. C. operate several times under a variety of circumstances since my return to this city, I am prepared to affirm that the utility of his method has even gained in my estimation. M. Lisfranc, whose authority in surgical matters is not to be despised, publicly remarked, the other day, that a surgeon was in his opinion a criminal who would not, whenever the thing is possible, resort to lithontrity; and that the knife should only be used when the first method failed, or when, from some peculiarities of the case, it could not be attempted. This strong language, and coming, as it does, from a man of M. L's high standing in surgery, is calculated to prove that M. C's. method (which on the whole appears to answer better than any other of the same kind) deserves to attract a little of our young men's attention when they visit Paris, and that it ought to be put in practice at home. M. LISFRANC, in proof of the decided advantage of lithontrity over the other operations for stone, remarked that, agreeably to some comparative tables made at Paris, it appeared that there generally died one out of five of patients cut in the various hospitals of that place; whereas, of about one hundred and fifty operations by the grinding method, its most bitter opponents had not been able to find out more than five or six fatal instances. And let it be remarked, moreover, that most of these fatal cases occurred in the hands of surgeons who were quite inexperienced in the use of the instruments." p. 20.

In another part of the same communication we are told, that the celebrated Scarpa of Italy, who had, at first, expressed an opinion unfavourable to the method of Civiale, has entirely withdrawn his opposition; and, finally, that in France there are, already, surgeons who devote themselves exclusively to the practice of Lithontrity.

We have chosen to present to our uninformed or sceptical readers, the testimony of these respectable and disinterested witnesses, in favour of the new method, rather than draw upon its author for proofs; and having, as we trust, said enough to inspire confidence, in that, to which we are converts ourselves, we shall proceed to say in what the new method consists.

The great object of our author, is to make known his lithontritic method, but his book contains much general information concerning urinary calculi, especially their physical characters and habitudes. The introduction, which runs through more than 50 pages, is devoted to a consideration of the different methods which have been proposed and practised, for ridding the bladder of calculi. These are *solution*, *extraction*, and *disintegration*, or breaking down. The difficulties and accidents of the two first (including a heavy mortality chargeable to extraction) are enumerated, in the spirit of one who believes in the great superiority of the last; and it is impossible to contemplate them, without feeling that a better method is desirable. Doubtless our author has presented them in the strongest light which the facts admit; but with all just allowance for the partiality of an inventor, it must be admitted, that multitudes have fallen victims to the gorget. Indeed, in the close of the article, our author expresses the opinion, that not a little concealment has been practised, in regard to the ill effects which have resulted from the cutting operation, lest patients should be deterred from submitting to it; a suppression of the truth which could not justly be condemned, as long as lithotomy was the only resource of the profession. In this country, however, the proportion of deaths from the operation, it appears to us, has been less than in Europe; a result more attributable, perhaps, to the bad air of *their* crowded hospitals, than to the superior skill of *our* lithotomists: among whom, however, there are not a few, who would distinguish themselves in any country.

The first chapter of Dr. Civiale's treatise is appropriated to the physical characters of urinary calculi, and to the causes which lead to their formation.

"Calculi differ in their *form*, their *density*, their *size*, and their *number*.

"In form they present a great variety, resulting from the elements of which they are composed, and the circumstances under which they are deposited. Calculi composed of the oxalate of lime, are almost always round and mammillary; those which consist of phosphate of lime and magnesia, have a less determinate form; but are generally round, or elongated and rough; those of lithic acid and lithate of ammonia, have in general an ovoid form, slightly flattened. When there are numerous calculi in the bladder, they, commonly, present a number of facets; and we see in many of these cases anomalies which are owing to particular circumstances.

"When the calculus fills the bladder, that organ serves as a kind of mould, which, in some degree, determines its form. We find, then, upon it two furrows, corresponding with the orifices of the ureters, along which the urine passes from those tubes.

"Calculi, the immediate causes of which, are foreign bodies, introduced, accidentally, into the bladder, have a form more or less similar to their nucleus. La Motte relates a case, in which the stone was formed around an iron wire. It was, says he, as long as the breadth of four fingers, and very slender.

"The hardness or density of calculi, presents differences which depend, not only on their composition, but also on their age. Those formed of oxalate of lime, are, in general, the hardest, except the siliceous, which are rare; those of lithic acid, are, generally, softer; those formed of the lithate of ammonia and the phosphate of ammonia and magnesia, have less density still; those composed of the phosphate of the lime, are, of the whole, the most friable. It should be observed, that all the varieties of calculi differ in hardness, according to the proportions of their constituent parts.

"We find some calculi a great deal harder in one part of their surface than another. Certain calculi are porous, and may be divided with great facility. In examining their fragments we perceive, that they are very hard and brittle. I have observed many facts of this kind.

"The size of calculi depends upon their age and their composition. They sometimes increase with a frightful rapidity. I have seen two patients, in whom calculi, in the space of six months, acquired the volume of walnuts. This rapid developement indicates with considerable certainty, that they are formed of the phosphate of lime, and are very friable. Calculi of this nature are susceptible of acquiring a greater volume than any others. Those of lithic acid and of the ammoniaco—magnesian phosphate, do not, in general, acquire so great a size, until they have lodged many years in the bladder. Those formed of oxalate of lime grow still slower.

"It is evident, that calculi can only be numerous, when they are small. In reference to their number, they present remarkable differences. Generally, we find only one or two; but Boerhaave informs us, that he saw even three hundred; a number that might appear incredible, if it were not justified by observations made in our own times. Mr. Murat, surgeon in chief of the Hospital of Bicetre, found six hundred and seventy-eight, in the bladder of an old man; and Dr. Beauchene found more than three hundred, in that of a man eighty years of age. We know, also, that upon the death of the celebrated Buffon, there were found in his bladder, fifty-five calculi of a triangular form. Flurant of Lyons extracted from a patient twenty-four calculi, of which sixteen had the volume of a pigeon's egg. Dr. Baneal, of Bordeaux, met in another patient, with twenty-eight of nearly the same size. Desault extracted more than two hundred from a priest of Pontoise; and I have taken sixteen from Mr. Thubeuf, Rector of a Nogent-le-Roi. In general when the bladder contains many calculi, they are composed of lithic acid. Mural calculi are commonly solitary, nevertheless we sometimes meet with many in the same patient. Mr. Maudhuyt, whose case I have reported, had two calculi, one of which was Mural." pp. 2—5.

Dr. Civiale thinks it imposible to determine what proportion of the inhabitants of any country, are affected with calculus; but it is possible to ascertain, with some certainty, at what age, and under what circumstances, they become affected with this malady. It appears that more than half who experience it, have not yet attained the age of puberty. According to the observations of Saucerotte, it is rare before the fourth year, but very frequent between the fourth and ninth; the proportion afterwards diminishes, to re-appear in a less degree between the fortieth and sixtieth, after which it decreases to the end of life. From the observations of the same surgeon it results, that the number of females affected with this malady, is to the males, as one to twenty four.

On the ætiology of calculus, our author says but little. It is most common in temperate climates, like our own. We are ignorant of the degree to which irregularities of regimen may be instrumental in its production. Excess of animal food is a frequent, and a sedentary life, a principal cause. Long continued retention of urine in the bladder, may be mentioned as another. But one of the most obvious, is the introduction of a foreign body through the urethra, which causes a deposition upon itself, of calculous matter. We recollect to have met with one case, in which a blow upon the lumbar region, causing temporary paralysis of the lower extremeties, was followed by the speedy developement of phosphatic calculi. An opinion is prevalent in the western country, that the use of limestone water favors the generation of calculi. Of the reality of this cause we have our doubts, but cannot at present enter into the inquiry.

The second chapter of our author's work is devoted to a consideration of the morbid influences of calculi upon the animal economy. The symptoms at the beginning are slight, and almost limited to the urinary system:—

"As the disease increases, the symptoms are greatly multiplied and aggravated, involving the functions of the body generally. The pains which were at first transient, become more constant and intense, extending to the perinæum, the groins, and even to the kidneys; a painful sense of weight is felt about the anus; the urine is glairy and fetid; its expulsion is frequent and increasingly painful: the slightest exercise augments all these symptoms; meanwhile the digestive functions become disordered; febrile movements manifest themselves, and the patient, if not relieved, falls into an alarming situation; the urine suffers greater vitiation, the pains become more excrutiating, and the fever more constant; the whole nervous system becomes seriously disordered, and the patient dies."

"Upon examination after death, the bladder presents traces of an inflammation, sometimes superficial, but oftener intense and carried to gangrene; its mucous surface is often deeply ulcerated, and its muscular tunic greatly developed, so that its parieties are thickened and applied closely to the stone. I have seen a calculus the asperities of which were implanted in them. In other cases, the organ acquires an unnatural capacity, when its parieties are attenuated, and we scarcely perceive a trace of inflammation. The neighboring parts partake more or less of the same pathological condition. The prostate gland is commonly indurated and sometimes in a state of ulceration, a blackish teint shows itself in the track of the urethra, especially, near the bladder; the ureters are enlarged, and present in their whole course, traces of inflammation; the kidneys are almost always affected; their volume is augmented and they present various alterations of tissue." pp. 8—10.

The third chapter is on the history of Lithontrity. It cannot be uninteresting to our readers to be told how the conception of this process, was excited in the mind of its inventor. Having long been distressed by the contemplation of the many cruel circumstances connected with lithotomy, he was led, in the year eighteen hundred and seventeen, into a course of unsuccessful experiments, for the dissolution of the stone in the bladder. While prosecuting these, it became obvious to him, that a preliminary measure, which should make him acquainted with the composition of every calculus, would be indispensibly necessary to the choice of an appropriate solvent. He was thus led to devise two instruments, by means of which he might seize the calculus, and detach from it such a portion as would enable him to determine its composition; it was the application of these instruments, that suggested to him the idea of mechanically breaking down the stone, so that it might be washed out of the bladder, and induced him to give up a chemical, for a mechanical method. The chapter contains a history of the progress of his inventions and discoveries, with an account of the various trials which preceeded the public annunciation of what he had achieved, but we shall not engage in its analysis.

The fourth chapter, a very short one, describes the urethra, as far as its anatomy is concerned in lithontrity. This organ may be divided into three sections—the first extends from the orifice to the bulb; the second from the bulb to the prostate gland; the third is surrounded by the gland:—

"The study of the last portion is of great importance in a practical point of view. Generally its direction, and its dimensions present no great variety; but they are subjected to changes from the diseases of the prostate." p. 49.

Chapter fifth brings us nearer to the main object of the book; and, indeed, treats of that which is the very foundation of the practice which it sets forth. An opinion is prevalent among the profession, that none but curved instruments can be introduced into the urethra; and if this were well founded, lithontrity could, obviously, have no existence. It is the object of this chapter to show, that *straight sounds and catheters may, with facility, be passed through the whole extent of the urethra*:—

"For a long time," says our author, "it has been thought impossible to penetrate into the bladder with straight sounds; and, therefore, these instruments were formed with a curve more or less adapted to that, which was attributed to the urethra. This point of surgical doctrine had received the sanction of ages, and even down to the present time, the introduction of a straight instrument was, generally, considered impracticable.

"Nevertheless straight sounds have been found in the ruins of Herculaneum; and the Arabian author Albucasis has transmitted to us drawings of such instruments." p. 52.

Among the moderns, this fact, so generally overlooked by the profession, was known to Lieutaud, Santarelli, Lassus and the authors of the Medical and Surgical Dictionary, published in 1772. But although straight sounds had long been known, very few rules for their use had been laid down; an omission the more to be regretted, as the manner of their introduction, differs essentially from that of crooked instruments. The following are our author's instructions for this purpose:—

"The surgeon should place himself on the right side of the patient, or between his legs. He must gently depress the penis, so as to bring it parallel with the thighs, which ought to be slightly flexed. The instrument, held in the right hand, is now to be introduced, and passes with facility to the symphysis pubis, which may be known by its resistence, and indicates, that we have reached the bulb of the urethra. Depressing the penis still lower, we direct the end of the instrument a little upwards, when it traverses the second or membranous portion of the urethra, and soon arrives at the prostate gland.

"Great attention to these rules is necessary, or else we may pierce the urethra, through the furrow that lies at the bulb, and carry the instrument even to the anus; an accident which has sometimes happened.

"The remainder of the operation requires still greater care. If the prostate gland is sound, it is sufficient, in most cases, to lower the hand, so as to direct the point of the instrument a little upwards, by which it will pass without difficulty into the bladder. But it is not the same when the gland is diseased, in reference to which it is not possible, however, to establish any general rule." pp. 54—5.

For exploring the bladder in search of a calculus, our author does not, however, recommend a *straight* sound. He thinks the ordinary curved instrument better adapted to the object, as it can be made to reach every part of the bladder. Of its introduction he does not speak, as being well known; and proceeds to make a few general remarks, two of which deserve to be translated.

"We ought," says he, "to prefer the sound to the catheter, as it may be introduced into the bladder when that organ is distended with urine, which often enables us to feel a calculus which cannot be found by the catheter.

"In some rare cases, we are obliged to vary the position of the patient, but we ought not to forget, that changes of posture are only useful when the bladder is full. It is in this manner, that we can determine by means of the sound if many stones exist." p. 57.

Our readers are now prepared, we trust, for a description of the instruments for lithontrity, with some occount of their application. The sixth and seventh chapters are appropriated to these objects, and we shall proceed to make such a digest of them, as may convey a clear idea of Dr. Civiale's method.

The apparatus consists chiefly:—

1. Of an exterior, straight, metallic canula, or sheath. For an adult it should be eleven inches long. Its diameter must vary from two to four lines. It may be made of silver, gold, platina, steel or copper—Dr. C. thinks silver the best. In operating, it is this part of the apparatus that is in contact with the urethra.

2. Of an interior tube, with one end slit down, and formed into pincers, called by Dr. C. *litholabes*. It must be longer than the other, and always made of steel. Its use is to seize and hold fast the calculus during the operation, and to withdraw the fragments when it is broken down. The pincers should consist of two, three or four branches; or rather the surgeon should be provided with several, adapted, by the number of their claws, to different cases and to the objects to be effected. They must be elastic, so as to expand when pushed beyond the inner extremity of the exterior canula; and their ends are flattened, rounded, and incurvated. They are not exactly of the same length, so that inclosing up they occupy, comparatively, but a small place, and form to the instrument a rounded head, which may be directed to any part of the surface of the bladder, with impunity to that organ. The pincers with two claws, may be used to extract small calculi from the urethra. Those with three branches, are preferred by the inventer for seizing, holding, and extracting the stone, in all ordinary cases. Those with four claws are only used under particular circumstances, which we need not enumerate. Sometimes one of the claws is moveable, and may be employed or removed at pleasure. The opposite, or outer extremity of the *litholabes* has a graduated scale which indicates the extent to which the claws are opened, and consequently the magnitude of the stone.

3. A perforating instrument, named by Dr. C. *lithotriteur*. It is this which, by a rotary or boring motion, penetrates the calculus and effects its disintegration. It is a solid steel rod, which is introduced through the hollow shaft of the *litholabes*, or pincers. Its head is armed with teeth, or projecting points, which act upon the calculus.

When the pincers are shut, this jagged extremity is so enclosed as to protect the bladder from its action. The other extremity is pointed, and, near the end, has a graduated scale, by which it may be accurately known how far the head of the instrument has perforated the stone. Dr. Civiale has devised two modes of augmenting the size of the aperture, made by the perforator:—1. by giving to the boring extremity a slight degree of curvature, by which it is made to describe, in the calculus, a much larger circle,—2. by forming the head of two parts, applied to each other longitudinally, and susceptible of being made to recede, so as to increase the diameter of the hole. Perforators of this kind are not so strong and solid as the others; but may be advantageously employed "when the stone is large, friable, and round."

Such are the implements which are introduced into the urethra and bladder. The remainder of the apparatus is composed of instruments, intended to connect these in their movements; to keep the pincers closed upon the calculus; to rotate the borer, and to project it forwards. Of this part of the machine it would be difficult to give an idea without the aid of the engravings, with which the book is illustrated; nor is a full description of them necessary, to the object which we have in view. They consist chiefly of a turner's lathe, in steel, the beam of which is six inches long. The permanent, or fixed puppet is to be screwed to the end of the outer canula, while the moveable, clasps firmly a leaden tube, open at one end, and containing a coiled steel wire, which projects an iron cylinder against the outer end of the perforator, and imparts to it a progressive motion. On the shaft of the perforator is a pulley, round which a turner's bowstring is passed, and thus a rotary motion is imparted by the hand of the surgeon.

The hollow shaft of the pincers, is prevented from passing into the bladder from the pressure of the borer against the stone, by a pressure screw, which passes through the outer tube, and by a button which is received against the external end of that canula, and which, at the same time, contributes to prevent the escape of the fluid from the bladder.

For the practice of lithontrity, our author, in his seventh chapter has laid down minute rules, of which we shall make but a brief abstract.

The operation may be performed at all seasons of the year. The patient must be prepared for it by an attention to the means of improving his general health, if it be impaired. If this even be good; he is to be subjected to a course of mild antiphlogistics; with a diet calculated to prevent inflammation. So much for the preparation of his general system. The urethra must be subjected to a special treatment, calculated to diminish its sensibility, and familiarize it to the presence of instruments. For this purpose, a flexible sound must be introduced, daily, for eight days or more, and kept in the urethra for ten minutes each time. Its size should be gradually increased, until the urethra will bear, without irritation, one that is equal in diameter to the exterior canula of the boring apparatus.

The patient being thus prepared, he is placed on his back with the pelvis raised, and warm water, or mucilage is injected into the bladder, through a canula. This instrument is then to be withdrawn, and immediately afterwards, the apparatus for the operation is to be introduced, in the manner already described for introducing straight sounds. The pincers are then to be expanded, and made to enclose and secure the stone. The hollow stem of the pincers is then to be immoveably fixed to the external sheath, or canula by a pressure-screw. The perforation may now be undertaken:—

> "In commencing, it is necessary to work slowly. If the stone is friable, the perforator enters with facility; and a dull sound is emitted: when it is hard, the sound is more acute, and the progress of the instrument is slower." p. 73.

The boring may generally be continued ten minutes. On withdrawing the apparatus, the first urine that flows is colored with blood. The patient is ordered immediately afterwards to take a bath, and to observe a mild regimen. By the next day he has in general, recovered from the operation, and is ready for its repetition; to which he commonly submits with less apprehension and greater confidence than before. The number of operations must depend on the size and hardness of the stone. The fragments are either discharged with the fluid contained in the bladder, or brought away by the pincers; and great pain should be taken to ascertain that they are all evacuated.

The eighth chapter, extending through 90 pages, is on the practice of lithontrity. It abounds in interesting cases, illustrating the operation, indicating the difficulties which may present themselves, and disclosing the circumstances which contra-indicate the method. It is impossible to read them without the conviction, that Dr. C. is a man of talents, caution and candour, whose claims to public confidence are of the highest order.

His cases and practical remarks, are divided into three series, or classes:—

"In the first series," says he, "I shall present only the cases of patients whose conditions were most favourable to the operation.

"I shall refer to the second, those in which the disease was far advanced, and offered complications which rendered the operation more difficult.

"Finally, in the third, I shall relate cases in which a successful resort to lithontrity was impossible." p. 76.

"The cases recorded in the first series, are not exactly similar; but in all of them the calculi had not attained to any great magnitude, or were few in number; the bladder was nearly sound; the urethra and prostate gland were healthy and well formed, and the general health of the patients was good. As our great object is to inspire confidence in the method of Dr. C. we shall translate the history of one of the cases referred to in this head, and without much selection, shall take one that was treated in the presence of a committee of the institute, and many other distinguished persons:—

"Mr. Gentil of Paris, aged 32 years, experienced for four years, a bad state of health, debility, impaired digestion and slight pains at the extremity of the penis. He entered the hospital of Caen, where they paid no attention to the last symptom. Sometime afterwards he returned to Paris; experiencing at all times more or less pain, and a sense of weight about the anus. On revisiting Caen, he was sounded by Dr. Dominel, who experienced some difficulty in introducing the instrument. He came again to Paris, and towards the end of November, 1823, applied to Mr. Dupuytren, who ascertained the existence of a calculus, and prescribed the carbonate of soda with gum arabic. The patient took these medicines for eight days; when he consulted Mr. Boyer. The presence of a stone being established, that surgeon proposed the operation of lithotomy. A few days afterwards the patient sought the advice of baron Larrey. That practitioner, likewise, recognized the existence of a calculus, but advised the operation to be postponed till spring. The patient was still dissatisfied, as the mere idea of an operation, was insupportable to him. Nevertheless, the violence of his pains did not permit him to remain at rest, and he consulted Moulin, who made him carry in the urethra a flexible sound for fifteen days; when, on the 9th of January, 1824, he applied to me.

"I judged that lithontrity was applicable, and the operation was commenced by me, five days afterwards, in presence of a commission appointed by the Institute, and of Messrs. Larrey, Sue, Giraudy, Moulin, Ponget, Nauches and others. The existence of a calculus being established, to the satisfaction of all who were present, the bladder was filled with warm water through the hollow sound, which was used in the examination. After having withdrawn the instrument, I introduced the apparatus, selecting a canula, three [illegible] diameter. I seized the the stone, of which the part embraced by the pincers was eleven lines in diameter, and proceeded immediately to its destruction. The first effect was so decided, that I supposed the calculus to be friable; but after some minutes the resistance which the perforator experienced, led me to think that the nucleus was harder than the crust.

"During this operation which lasted twenty minutes, I fixed and attacked the calculus in three different positions. I then made a second injection through the exterior tube of the perforator, and the fluid in returning brought with it a considerable quantity of detritus or powdered stone, and some small fragments. On seeing this result Percy exclaimed, "*Gentlemen behold the proof positive!*" The sufferings of the patient were slight. Towards the end of the operation he compl ined of being a little unwell, but manifested more weariness than pain; and half an hour after it was terminated, stepped into his carriage and rode home. Next day he had a slight paroxysm of fever, which went off with a gentle perspiration; his tongue was a little furred; he had experienced no disorder in the urinary passages; and had rendered a quantity of stony powder with his urine. On the following days his condition presented nothing worthy of particular notice.

"Six days afterwards I resumed the operation, in presence of the same practitioners, and of Messrs. Magendie, Serres and Aumont; and had the satisfaction to obtain the same happy results, as at the first operation; some voluminous fragments were detached, which I extracted with the pincers, which seized them with great facility.—This operation continued near thirty five minutes; the patient suffered very little, and discharged with his urine a quantity of *detritus* and some small fragments. The next day after the operation, and the following days, no particular symptoms supervened: hip baths, enemata, and emollient drinks, were continued for nearly a week.—A third operation was then instituted, which only continued twenty minutes. New fragments were detached; and by the aid of an injection a considerable quantity of powdered calculus was brought away. The sufferings of the patient were still less in this trial, than the last; because it was shorter; and in a quarter of an hour he was able to enter his carriage. There only remained in the bladder a single fragment too large to be expelled with the urine, it was broken with the pincers, and discharged a few days afterwards. One of its pieces was very irregular, weighed ten grains, was six lines and a half long, and eleven lines and a half in circumference. From the moment it was discharged, the patient experienced great relief, and his strength returned daily. I have since sounded him many times, and am convinced that the cure is perfect. The treatment did not continue a month; the patient was at no time confined to bed; and there were only three paroxysms of fever induced by the long operations which were practised, and by all my efforts to extract the fragments. Baron Larrey sounded him two years after the operation, and ascertained that he had nothing in the bladder." pp. 82—86.

Notwithstanding the length of this case, we are tempted to extract another, of a more recent date, which presents the value of the *Methode Civiale*, in a still more encouraging point of view:—

"Mr. Bourla of Brest, aged 19, had laboured under calculus for ten years. I ascertained that it was mammillary and of considerable size. Afterwards, I discovered that it was mural, but nevertheless friable. The first attempt with the lithontriter was made on the 27th of May, 1825. I experienced some difficulty in seizing the stone on account of its size; and should have felt that there was a difficulty in fixing it, if the inequalities of its surface, had not convinced me that it might be held as it was seized, so I proceeded to the operation.

"During a month, Mr. Bourla underwent nine operations, which did not present any circumstances worthy of particular notice: after each one, numerous fragments were discharged with the urine.

"He suffered so little during the whole time, that he walked to and from my house, to be operated on.

"Although extremely sensitive, he did not experience any of the accidents that might have been apprehended in such a temperament; and acknowledged with a smile, that he had suffered more from fear han pain.

"I have this moment received a letter from him—written after returning from a long voyage—in which he assures me that his health is excellent.

"The treatment of this case was conducted in pressence of his Excellency the Swedish Ambassador, of Messrs. Arago, Thenard, Geoffroy St. Hiliare, Vauquelin, Vigaroux, and many other distinguished practitioners of the Capital." p. 123.

Now, if by the method of Civiale, a large stone of ten years duration, could be destroyed and removed in a month, without pain or fever, and the patient go abroad throughout the whole period, ought not that method to be introduced into every country? Why should it not make an essential part of the studies of every young man, who dedicates himself to surgery? It certainly ought.

We come to the second and third sections of this chapter, the subjects of which we have already announced. Cases referred to these heads, do not of course resemble each other so closely as the first; seeing that the circumstances which render the operation difficult, or impracticable, are necessarily much diversified.

Strictures of the urethra, and excessive sensibility of that tube; a similar state of the vital properties of the bladder; chronic cystitis; organic changes in the parieties; diseases of the prostate; the multiplication of calculi; and a bad state of the general health of the patient, are circumstances that render the operation of lithontrity difficult and dangerous. It should be regarded as impracticable or as contra-indicated;—when the disease has lasted for so long a time, that the calculus has acquired great dimensions, and the bladder, contracting firmly upon it, has become horny; when the calculi are numerous and of considerable size, and the health of the bladder, and that of the general system is bad; generally, when the stone is encysted; when it is large and lodged in the urethra; when that tube from *organic* changes has become, in a great degree, impervious; when the nucleus of the stone, is a foreign body of such a kind, that it cannot be disintegrated; finally, when the kidneys are in a state of organic disease. Many of these conditions render lithotomy not less than lithontrity, hazardous, impossible, or fatal; Dr. C. has, however, reported some cases in which, after a reluctant trial of his method, he was induced to desist, and the patients were afterwards cured by the old operation.

Lithontrity would seem, at first view, to be much better adapted to females than males; but although the great diameter, shortness, and slight curvature of the urethra, are favorable circumstances; Dr. C. has found, that it is difficult to introduce the instrument into the *meatus urinarius;* when once introduced, however, the destruction of the calculus and its extraction are readily effected.

The ninth chapter of Dr. Civiale's book, is devoted to the objections that have been started to his method. He has replied to them in a resolute spirit; and sustained the practice, by the citation of a great number of cases. Our limits will only permit us to notice the practical objections, with his answers, and this in the briefest manner:—

"Any sudden change in an essential part of the art of cure, must necessarily combat ancient practices and habits; we should not be astonished, then, that lithontrity, which professes, in a great majority of cases, to render one of the most difficult and important operations of surgery unnecessary, should have met with opposition. The objections which have been made to my method are generally known, nevertheless, I shall enumerate them in this chapter, and show that most of them are unfounded:—

"The possibility of penetrating into the bladder with straight instruments has been denied. On this subject I need not add any thing to what I have already said in my 5th chapter." p. 167.

"The difficulty and danger of dilating the urethra, so far as to admit my instruments, is another objection; but those who know the structure of that canal are aware how easy it is to introduce sounds, of from two to four lines in diameter. Now the greatest diameter of my instruments is but four lines; and those which I commonly employ are but three. they do not, therefore, cause any forcible dilatation of the canal. When there is a stricture, it is sufficient, by the ordinary means, to restore the urethra to its natural size; and the cure of that affection becomes, then, the preparatory treatment for lithontrity." p. 168.

"Fears have been expressed relative to the strength and solidity of my instruments; but experience has demonstrated, that these are unfounded. p. 170.

"A great deal has been said on the danger to the bladder, from the pincers which I employ; it is only necessary, however, to glance the eye upon the plates which represent them, to be convinced that this danger is imaginary. The bladder cannot be pinched when it is full of water, and we operate with ordinary care." p. 171.

"Some surgeons have thought that the practice of lithontrity presented very great difficulties. Without doubt, there is some difficulty in seizing the stone, but the operator soon acquires the necessary experience.

"The pain attendant on the operation has been made another objection, and the reports concerning this have been greatly exaggerated. Experience has proved, that they are generally slight; and if in some cases they are severe, they are in none equal to what the patient has often experienced in the course of the disease. In a great nnmber of instances no fever is excited, and the patient can walk about immediately after the operation." p. 171.

We may here state in support of the assertion of our author, that when Dr. Brown was lately in Paris, he was present at an operation on a man nearly 60 yea s of age, who, immediately after it was done, walked a mile with Dr. Brown, and conversed cheerfully the whole way.

"My method, it has been said, is only applicable to a certain number of cases. Now the cases recorded in this work can leave no doubt that the greatest, I might say the only obstacles, to the operation, has been the large volume of the stone, and the organic changes in the bladder, resulting from the long continuance of the disease. But this great duration has been occasioned by the fear of the knife, which induces patients to postpone a resort to lithotomy. My method presents none of these terrors, and the patients may, therefore, submit to it in the early stages of the disease, when it must generally be successful." p. 175.

"The possibility of relieving the bladder from every fragment of the stone, by lithrontity, has been strongly contested. But, for the three years that I have practised it, I have kept up an intercourse with all my patients, and the disease has not been re-produced in any of them." p. 178.

In addition, Dr. Civiale has reported several cases, in which death took place from different causes, sometime after the persons had been subjected to lithontrity, and by autopsic examinations it was ascertained, that the bladder was entirely free from foreign bodies.

"A state of nervous irritation must, as has been urged, be unfavorable to the employment of my method; but if the stone be small, and appropriate modes of calming the irritation of the system be pursued, the operation will be found practicable." p. 187.

"Some surgeons have thought, but incorrectly, that engorgements of the prostate gland might prevent the employment of lithontrity. The difficulty which they oppose to the introduction of straight instruments, may, in general, be surmounted by means of the precautions which I have already laid down; and they are not, perhaps, in any case sufficient to make us renounce the operation." p. 195.

"The state of the urinary organs before the time of puberty, must, say the objectors, oppose an obstacle to the lithontritic method, in children. But, in a great number of cases, I have operated successfully with an instrument only two lines in diameter; and such may be used with children after the third year." p. 230.

"The introduction of a straight sound, in children, is not so difficult as we might think, from the anatomical disposition of the parts. The great curvature of the urethra is easily overcome, and the prostate gland presents no difficulties." p. 206.

Finally, the objection to Dr. Civiale's method, on which the greatest stress has been laid, and which has been most frequently repeated, is, that it cannot be practised, when there is a stricture of the urethra. This, however, is no objection to it when strictures do *not* exist; and they are absent in a majority of cases. We have already quoted our author's remarks on this subject. The removal of the stricture, is the step which prepares the patient for the operation. To this point he has paid particular attention. He has invented and delineated a set of instruments for the purpose; and his tenth and last chapter, details his methods of cure. As we intend, however, in an early number, to make strictures of the urethra the subject of a separate article, we shall not, at present, disclose the views and practice of Dr. Civiale.

The appendix to the book contains a tabular view of the cases of calculus, treated by on the plan of Dr. Civiale, which we shall present to our readers entire, as affording connected data for an estimate of its value.

### ANALYTICAL TABLE.

*Of operations performed by Dr. Civiale, for breaking down the stone in the bladder. Presented in the order in which they were executed.*

| Nos. | Dates. | names and ages of the patients. | Duration of the disease, | Duration of the treatment | OBSERVATIONS. |
|---|---|---|---|---|---|
| 1 | 1823 | Boulbar, 64 years, | 2 months, | 18 days. | The disease was complicated with a suppression of urine, occasioned by a stricture of the urethra, the treatment of which served to prepare the patient for the operation. |
| 2 | —— | N——, 40 years. | 3 months, | 8 days. | The stone small and iable. It was broken and extracted in one operation, which continued five minutes. I did not submit the patient to any prepartion. |
| 3 | 1824 | Gentil, 30 years, | 3 years, | 1 month, | The stone mural (mulberry) and of the size of a large walnut. Its great hardness rendered the operation tedious; but it was perfect. |
| 4 | —— | Laurent, 40 years, | 6 months, | 1 month, | The stone friable, and of the size of an ordinary walnut. It had for its nucleus a French bean, which was extracted with the fragments of the stone. The irritability of the bladder rendered the operation difficult. |
| 5 | —— | Perot, 30 years, | 18 months, | 1 month, | Two hard calculi, of the size of a large almond. The small diameter of the urethra, and the irritability of the bladder, were opposed to the operation. Some imprudences of the patient caused a swelling of the testes, and retarded the cure. |
| 6 | —— | Lebaigue, 66 years, | 4 years, | 1 mo. & a half | Two very hard calculi of the size of a small hens egg; nevertheless a favourable conformation of the parts, rendered the operation easy. Six months after the cure, Labaigue fell a victim to an affection of the right kidney. An autopsic examination established this fact, and also, proved, that the bladder was sound, and did not contain a single fragment of calculus. |

| | | | | | |
|---|---|---|---|---|---|
| 7 | — | Boutin, 36 years, | 3 years, | 1 mo. & a half | Stone very hard, flattened, oblong, and of the size of a walnut. Its form augmented the difficulty of the operation. Many paroxysms of fever, and other causes foreign to the operation, prolonged the duration of the treatment. |
| 8 | — | Cortial, 56 years, | 3 years, | 3 months, | Stone friable and very voluminous. Engorgement of the prostate, considerable. The bladder in a decidedly unhealthy state. Operation extremely difficult. Many of the first trials were unsuccessful. Symptoms of gravel afterwards manifested themselves slowly. At a subsequent time two small stones were extracted. |
| 9 | — | Maud'huyt, 40 years, | 4 years, | 15 days, | Two calculi of the size and form of an almond; one mural and very hard—the other more friable. The patients disease had been misunderstood for many years, and his health had suffered from it. |
| 10 | — | Cornu, 72 years, | 5 years, | 3 months, | Two voluminous and very hard calculi. The bladder unhealthy; the constitution very feeble. Before the treatment was finished, Mr. Cornu, from an irregularity of living, experienced an attack of acute gastritis, from which he died. Examination after death disclosed what we have just stated; and, besides, that there still remained in the bladder a fragment of stone an inch and a half long in its greatest diameter. The organ itself was in the state in which we generally find it, in cases of long standing. |
| 11 | — | Perin LePage, 45 ys. | 15 months, | 18 days, | Calculus friable, and of the size of a small walnut. The operation easy. |
| 12 | — | Mrs. DeLange, 72 ys. | 18 months, | 12 days, | The calculus small and friable. The operation did not present any difficulty. The extreme weakness of the patient did not constitute an obstacle to her cure. |
| 13 | — | Thubeuf, 66 years, | 3 years, | 3 months, | Sixteen calculi of which fifteen had the size of a hazlenut; while the other was much larger. The prostate much engorged: the bladder very irritable. The operation extremely difficult. A nephritic colic, to which the patient was liable, required us to suspend the treatment for a month and a half. |

| | | | | | |
|---|---|---|---|---|---|
| 14 | —— | Remond, 65 years, | 2 years, | 1 mo. & a half | The stone hard, and of the size of a small hen's egg. The great irritability of this patient, and his predisposition to apoplexy, required me to proceed with caution; and diminished the chances of success. |
| 15 | 1825 | Azille, 65 years, | 18 months, | 21 days, | Stone hard; of the size of a walnut. Up to the day of the operation Mr. Dupuytren had not been able to find this stone with the common catheter; by means of my instrument it was at once discovered. The operation did not present any difficulty. A fragment remained in the bladder which kept up the pains; it was extracted and the cure became complete. A year afterwards the patient died of a cancerous affection of the pylorus. |
| 16 | —— | Desrotours, 50years, | 18 months, | 1 month, | Stone very hard, oblong, flattened, from 12 to 15 lines, in its longest diameter. Some difficulties caused by the form of the stone, presented themselves in the beginning of the treatment. The operation afterwards became easy. |
| 17 | —— | Erard, 73 years, | 4 years, | 2 mo. & a half | Many very hard calculi. A bad state of the patient's health, with a marked predisposition to congestion of the lungs, rendered the success of the operation doubtful, and prolonged the treatment. |
| 18 | —— | Desprets, 45 years, | 4 years, | 1 month, | Two hard calculi, of the size of an almond. The operation easy. |
| 19 | —— | Balet, 68 years, | 2 years, | 1 mo. & a half | Many friable calculi. A severe affection of the chest and a bad state of the bladder, detracted much from the prospect of success; nevertheless the patient was cured of the stone. A year afterwards, he sunk under a pulmonary affection. Before his death he had commenced discharging gravel. |
| 20 | —— | Bourla, 19 years, | 10 years, | 1 mo. & a half | The stone mural, (mulberry) of the size of a large hen's egg. Repeated operations, but easily performed. |
| 21 | | Brousseaud, 45 years | 1 year, | 1 month, | Many small and hard calculi. An engorgement of the prostate gland rendered the operation difficult. |
| 22 | —— | Huet, 56 years, | 3 years, | 1 month, | The stone large, but friable. A catarrh of the bladder, an asthmatic affection, and an aneurism of the heart, rendered the operation very hazardous: nevertheless it succeeded. A month afterwards, the aneu- |

| | | | | | |
|---|---|---|---|---|---|
| | | | | | rism caused the death of the patient. A *post mortem* examination established this fact, showed that the bladder was sound, and that the stone was entirely removed. |
| 23 | —— | Leclere, 55 years, | 3 years, | 1 month, | Many small calculi. The patient very irritable and extremely weak, which prolonged the treatment. |
| 24 | —— | Fournier, 60 years, | 18 months, | 22 days, | Many very hard calculi. The operation easy; but an old malady of the chest became much worse. About two months afterwards the patient died of a neglected suppression of urine. |
| 25 | —— | Guilbert, 35 years, | 3 years, | 25 days, | The stone friable, and of the size of an ordinary walnut. Irritability excessive. Operation easy. |
| 26 | —— | Matre, 40 years, | 4 years, | 1 mo. &a half | The stone large and hard; the bladder unhealthy; the prostate engorged; the operation difficult. |
| 27 | —— | Dauza, 66 years, | 2 years, | 3 months, | Stone voluminous and friable. Paralysis of the bladder, incontinence of urine, œdema of the lower extremities, asthma, continued fever. These severe complications, in a great degree, disappeared after the operation. |
| 28 | —— | Travers, 75 years, | 3 years, | 1 month, | Stone of the size of a walnut. An engorgement of the prostate, irritability of the bladder, and the great age of the patient, were not obstacles to the success of the operation. |
| 29 | —— | Lucotte, 56 years, | 2 years, | several mo. | Many calculi. The prostate very large. Irritability of the bladder carried to the highest degree. Different circumstances, foreign to the operation, compelled me to suspend it, which prolonged the treatment for several months. The operation presented great difficulties. |
| 30 | —— | Belin, 36 years, | 15 months, | 15 days, | The stone friable; it had for a nucleus a beard of grain and a piece of straw, which were extricated with the stone. The operation easy. |
| 31 | 1826 | Fichon, 40 years, | 1 year, | 8 days, | Stone small. Was broken in seven minutes. Some fragments were extracted with the pincers, the remainder came away with the urine. |

| | | | | | |
|---|---|---|---|---|---|
| 32 | — | Champanhac, 60 ys. | 3 years, | 1 mo, & a half | Stone hard; of the size of a walnut. Bladder unhealthy; prostate engorged. Operation difficult. |
| 33 | — | Viallanes, 58 years, | 1 year, | 20 days, | Many small friable calculi. Operation easy. Three months after the operation, the patient fell a victim to a malady which had no connexion with it. This fact was established by an autopsic examination; which also assured us, that the bladder was sound, and did not contain any fragments of stone. |
| 34 | — | N———, 32 years, | 2 months, | 12 days, | Two calculi; one was fastened in the urethra and extracted, the other broken in the bladder. The operation very easy. |
| 35 | — | Galle, 13 years, | 6 years, | 2 months, | Two calculi of the size of a walnut, and very hard. The necessity of employing an instrument of the smallest diameter, rendered the operation difficult, and prolonged the duration of the treatment. |
| 36 | — | Mourot, 66 years, | 14 years, | 2 months, | A great number of very hard calculi, some of them as large as a walnut. A favourable conformation of the parts, facilitated the operation by enabling me to employ an instrument of four lines—the largest that I have used. |
| 37 | — | Seve, 40 years, | 18 months, | 1 mo. & a half | A stone of the middle size. The great irritability of the patient, compelled me to operate but seldom, which prolonged the treatment. |
| 38 | — | Martin, 43 years, | 5 months, | 25 days, | Stone hard; from 12 to 15 lines in its diameter; the operation easy. |
| 39 | — | Anthoine, 45 years, | 6 years, | 1 month, | The stone friable; of the largest size that I have ever broken; nevertheless the operation was easier than might have been expected. For 18 months the patient had labored under an incontinence of urine; it ceased after the operation. |
| 40 | — | Oudet. 60 years, | 18 months, | 21 days, | Stone of a middle size. Operation easy. |
| 41 | — | Courtois, 60 years, | 3 years, | 18 days, | Stone voluminous. No difficulty in the operation. |
| 42 | — | Michel, 41 years, | 14 months, | 1 month, | Many small calculi; the bladder very irritable, with great organic disease. The operation easy, |
| 43 | — | Bousquet, 63 years, | 15 months, | 1 month, | A small blackish calculus, porous and brittle. Ordinary sounding had furnished but very uncertain *data*. On the first exploration with my instrument, the stone was immediately found, seized and divided. The operation did not offer any difficulty. |

"All the patients on whom I have operated, with one exception, were cured. Those who died sometime after the operation, fell victims to causes entirely foreign from it. In the majority of these cases, it was ascertained by dissection, that the bladder was sound, and did no tcontain any fragments of stone."

With these extracts we close our analysis. If our readers have thought it tedious, they must remember the novelty, magnitude and importance of the subject. A subject of deep interest to the profession, and, as yet, but little understood, or regarded in America. Our object is to make it known, at least in outline. and to invite our surgical brethren to investigate it. To do this, we have thought it necessary to lay before them, as many facts as our limits would permit, and confidently hope, that we have not laboured in vain. For ourselves, we see no reason for doubting what Dr. Civiale has published. His experiments have not been made in secret; but, as the history of his cases informs us, under the scrutinizing observation of the most distinguished surgeons of the French metropolis, than whom, the world could not furnish more competent judges.

That the operation of *lithontrity* cannot be successfully performed without laborious preparation, is quite probable; but this constitutes no objection to it, when we recollect, that the operation of *lithotomy*, requires a long course of study and experience to render *it* successful.

In point of danger, the two operations seem to us scarcely to admit of a comparison—although one is yet in its infancy, and the other has for ages been advancing to its present condition. As to the pain which they inflict, the contrast, if we credit either theory or experience, is equally striking. Finally, in the terror with which they agitate the unfortunate persons who are compelled to resort to one or the other, the difference must be equally in favour of the *methode Civiale.*—We trust, therefore, that the time is not distant, when a competent number of our professional brethren, will be found seriously engaged, in dispensing, to their afflicted countrymen, the benefits which it seems so well qualified to confer.

We shall conclude by adopting the eloquent language of

Messrs. Chaussier and Percy, who, in their report to the royal Academy of Medicine, on the invention of Dr. Civale, pronounce it "EQUALLY HONOURABLE TO ITS AUTHOR, GLORIOUS TO FRENCH SURGERY AND CONSOLING TO HUMANITY."

